# Sensorimotor Oscillations During a Reciprocal Touch Paradigm With a Human or Robot Partner

**DOI:** 10.3389/fpsyg.2018.02280

**Published:** 2018-12-10

**Authors:** Nathan J. Smyk, Staci Meredith Weiss, Peter J. Marshall

**Affiliations:** Department of Psychology, Temple University, Philadelphia, PA, United States

**Keywords:** human–robot interaction, mu desynchronization, beta synchronization, social robotics, tactile perception

## Abstract

Robots provide an opportunity to extend research on the cognitive, perceptual, and neural processes involved in social interaction. This study examined how sensorimotor oscillatory electroencephalogram (EEG) activity can be influenced by the perceived nature of a task partner – human or robot – during a novel “reciprocal touch” paradigm. Twenty adult participants viewed a demonstration of a robot that could “feel” tactile stimulation through a haptic sensor on its hand and “see” changes in light through a photoreceptor at the level of the eyes; the robot responded to touch or changes in light by moving a contralateral digit. During EEG collection, participants engaged in a joint task that involved sending tactile stimulation to a partner (robot or human) and receiving tactile stimulation back. Tactile stimulation sent by the participant was initiated by a button press and was delivered 1500 ms later via an inflatable membrane on the hand of the human or on the haptic sensor of the robot partner. Stimulation to the participant’s finger (from the partner) was sent on a fixed schedule, regardless of partner type. We analyzed activity of the sensorimotor mu rhythm during anticipation of tactile stimulation to the right hand, comparing mu activity at central electrode sites when participants believed that tactile stimulation was initiated by a robot or a human, and to trials in which “nobody” received stimulation. There was a significant difference in contralateral mu rhythm activity between anticipating stimulation from a human partner and the “nobody” condition. This effect was less pronounced for anticipation of stimulation from the robot partner. Analyses also examined beta rhythm responses to the execution of the button press, comparing oscillatory activity when participants sent tactile stimulation to the robot or the human partner. The extent of beta rebound at frontocentral electrode sites following the button press differed between conditions, with a significantly larger increase in beta power when participants sent tactile stimulation to a robot partner compared to the human partner. This increase in beta power may reflect greater predictably in event outcomes. This new paradigm and the novel findings advance the neuroscientific study of human–robot interaction.

## Introduction

As automation and technology become more ubiquitous in society, it is increasingly commonplace for interactions that have typically occurred between humans to also occur between humans and robots. Social-cognitive neuroscience offers a novel window into these interactions. Robots are traditionally constructed as highly complex tools, a design approach that persists in the present discourse on robotics and society (Šabanović, 2010). Increasingly, robots are designed as social agents, capable of interacting with humans in varied natural settings (Fong et al., 2003). Social and interactive robots have been developed for healthcare applications (Wada and Shibata, 2007; Broadbent et al., 2010; Robinson et al., 2014; Mann et al., 2015), educational settings (Tanaka et al., 2007; Toh et al., 2016), and mental health treatments (Begum et al., 2016). Social robots designed for these domains are often embodied, with varying degrees of human likeness; there is evidence that embodied social agents are more judged more favorably than disembodied social agents (Lee et al., 2006), especially within the context of social touch (Cramer et al., 2009a,b). Disembodied robots may either be simulated via a computer program or presented remotely through a screen. In either case, people empathize more with robots when they are physically embodied and present, compared with agents that are disembodied (Kwak et al., 2013). Given the embodied nature of social robots, it is likely that our interactions with such machines will increasingly involve tactile experiences (Huisman et al., 2013).

Humans use touch to communicate a wide range of social and emotional information (Knapp et al., 2013), and there is a good deal of current interest in this channel of communication in the context of human–robot interaction (HRI) (Gallace and Spence, 2010; Van Erp and Toet, 2015). We suggest that methods from social cognitive neuroscience can be applied to questions within the field of HRI, informing the design of robot entities to make human engagement with technology more fluid. Additionally, robots provide an opportunity to study human social behavior in various ways (Broadbent, 2017). There are many factors affecting how we perceive robots, from physical organization and appearance, to more subtle influences based on function and perceived intent. Functional affordances of a robot are the actions it is able to do, be it physical actions, gestures, or utterances (Awaad et al., 2015). In a study examining responses to multiple kinds of social robots, participants were more likely to report stronger engagement with a robot and intention to use it if it had sufficient affordances to complete a physical task, while physical appearance was rated as less important for engagement (Paauwe et al., 2015). Humanoid social robots with the ability to communicate through arm and hand gestures are rated as more anthropomorphic and likeable than physically identical robots without these capabilities (Salem et al., 2013).

Behavioral research within HRI has uncovered multiple factors and contexts that influence the ways in which people interact with and perceive robotic agents, specifically in the context of touch. Participants in a collaborative virtual reality environment rated a virtual agent capable of social touch through vibrotactile feedback more positively on affective adjectives than they did a non-touching agent (Huisman et al., 2014). Touch to (and from) a robot was shown to encourage participants’ effort on a simple motor task (Shiomi et al., 2017). More specifically, active touch from a robot has been shown to be a stronger motivator than passive touch (Nakagawa et al., 2011). When interacting with robotic hands, participants report increased feelings of trust and friendship when the hands are warm, compared to cold robot hands or holding no hand at all (Nie et al., 2012); a similar study with a robotic social dinosaur found people liked warmer versions compared to tepid or cold conditions (Park and Lee, 2014).

One goal of research in HRI is to quantify whether robotic partners are sufficient analogs for human contact across different domains; perhaps unsurprisingly, there are contexts in which people prefer the contact of humans. In a study conducted in a nursing home, patients were comfortable with a robot touching their arm when they believed the robots intention was to clean them, but responded less positively when they believed the robot intended to provide them comfort through touch (Chen et al., 2014). A robot completing menial physical tasks fits well within our conception of what robots ought to do, but people tend to have reservations when imagining robots in social roles (Fong et al., 2003). In individuals with preexisting negative feelings toward robots, physical contact was shown to increase those negative attitudes (Wang and Quadflieg, 2015; Wullenkord et al., 2016); the inverse was true for those with positive attitudes. While a massage is both a functional and social task, participants receiving a head massage from a robot rated it worse than an equivalent massage delivered by a human (Walker and Bartneck, 2013). There is evidence that people may feel arousal or embarrassment when asked to touch the intimate parts of a robot (Li et al., 2016), and that humans also feel they are able to convey comforting and affectionate emotional states to a haptic machine through the action of touch (Yohanan and MacLean, 2012). These examples serve to highlight the complex nature of touch between humans and robots, and the many ways it differs across context and application.

Beyond the preceding review, little research exists on how humans respond to tactile interactions with robots, and even less work has considered the neural processes associated with these interactions. There is a robust literature within social cognitive neuroscience on the neural underpinnings of social touch, and it is the goal of the present study to combine those approaches with the goals and methods of HRI. Our interest lies in how examining neural activity related to touch can inform the psychological differences in interactions with robots rather than humans. To probe this question, we investigated whether oscillatory neural activity in anticipation of receiving tactile stimulation is influenced by participants’ beliefs about whether the tactile stimulation was initiated by either a human or a robot agent. We were also interested in whether sensorimotor brain potentials associated with triggering tactile stimulation to another entity differ when the entity is a human or a robot. To examine these questions we employed a novel “reciprocal touch” paradigm and utilized measures derived from the electroencephalogram (EEG), specifically the sensorimotor mu and beta rhythms. Within the domain of social cognitive neuroscience, these rhythms have often been studied in the context of the connections between the actions of the self and the actions of others, including tactile aspects of these linkages (Marshall and Meltzoff, 2015; Shen et al., 2017). Combining this line of work with behavioral insights from HRI research can help to forge new directions in the study of human responses to interacting with machines.

Changes in EEG brain rhythms have proven useful as reliable indicators of attentional orienting to touch, predicting perception of a subsequent weak tactile stimulus when that stimulus can reliably be expected (Zhang and Ding, 2010). Recent work in this area has focused interest on alpha-range rhythms, particularly in relation to anticipatory attention. Anticipatory desynchronization of alpha oscillations appears to be an index of local sensory cortex excitability, with heightened desynchronization associated with the perceptual salience of upcoming stimuli (Zhang and Ding, 2010; Foxe and Snyder, 2011). While this phenomenon has been studied in various sensory modalities, the focus here is on the sensorimotor mu rhythm in the EEG in relation to touch. The mu rhythm is an alpha-range oscillation that occurs at 8–13 Hz in adults and is typically observed over central electrode sites. During anticipation of impending tactile stimulation of the hand, there is a reduction of mu rhythm amplitude over contralateral somatosensory cortex (Anderson and Ding, 2011; Haegens et al., 2012; Shen et al., 2017). This desynchronization of the mu rhythm appears to reflect an increase in local field potentials of neurons in somatosensory cortex (Gomez-Ramirez et al., 2016). Shen et al. (2017) observed mu desynchronization in anticipation of tactile stimulation to one’s own hand, which was not present when a partner or “nobody” received tactile stimulation. However, it is unknown whether the perceived origin of tactile stimulation delivered to the self (e.g., tactile stimulation initiated by a human vs. a machine) influences the amplitude of mu rhythm modulation during anticipation of touch.

While much research on the mu rhythm has concerned anticipatory attention, the EEG beta rhythm (14–30 Hz) has mainly been examined in the context of action production (Puzzo et al., 2011). Beta rhythm responses are modulated by motor movement and imagery (McFarland et al., 2000), and appear to reflect various spatial and temporal attentional mechanisms (van Ede et al., 2011). The beta response to the initiation of movement of the hands has been localized to the contralateral sensorimotor cortex, and takes the form of an event-related desynchronization (ERD) (Miller et al., 2007) followed by an event-related synchronization (ERS) that appears to reflect activity around the precentral gyrus (MI) (Gaetz and Cheyne, 2006). The increase in beta amplitude (i.e., beta rebound) following motor movement initiation is believed to reflect decreased cortical excitability and reduced processing of afferent sensory information involved in motor feedback (Pfurtscheller, 2001), and is also related to greater predictability of events and maintenance of the sensorimotor set (Engel and Fries, 2010). Alpha and beta oscillatory responses have also been widely used in the development and implementation of brain-computer interfaces (Yuan and He, 2014), with particular interest in the beta rhythm due to the range of human behaviors that engender this rhythm; the beta rhythm is responsive to both overt and imagined motor movement, and can be used to control machines (Neuper et al., 2009).

The present study introduces a novel paradigm in which participants carried out a joint tactile task with a robot or a human partner. The study aimed to answer the following questions: (1) Are different anticipatory neural responses seen to impending tactile stimulation to the self if it is believed that this stimulation is initiated by a human versus a robot partner?; (2) Is the sensorimotor EEG response to initiating tactile stimulation different when the target of the stimulation is a human or robot partner? In order to address these questions, we collected EEG from adult participants while they engaged in a turn-taking task with a robot or human partner. The overarching aim of the study was to contribute to the development of new HRI protocols in which human brain activity is monitored during interaction with robotic agents in a controlled setting.

## Materials and Methods

### Participants

Twenty undergraduates (18 females; mean age = 19.70 years; *SD* = 2.34) received course credit in return for participation. This study was carried out with approval from the Institutional Review Board at Temple University, with informed consent being obtained from each participant. All participants were right handed according to the Oldfield handedness questionnaire (Oldfield, 1971), had normal or corrected vision, and reported no history of neurological illness or abnormality.

### Stimuli and Materials

#### Tactile Stimulation

Tactile stimuli were delivered using an inflatable membrane (10 mm diameter) mounted in a plastic casing and attached to the finger by a flexible plastic clip. The membrane was attached to the right index finger of the participants and their human partner; the membrane was attached to the haptic sensor of the robotic task partner (see below). The membrane was inflated by a short burst of compressed air delivered via flexible polyurethane tubing (3 m length, 3.2 mm outer diameter). The compressed air delivery was controlled by STIM stimulus presentation software in combination with a pneumatic stimulator unit (both from James Long Company) and an adjustable regulator that restricted the airflow to 60 psi. The pneumatic stimulator and regulator were located in an adjacent room to the participant. To generate each tactile stimulus, the STIM software delivered a TTL trigger (10 ms duration) that served to open and close a solenoid in the pneumatic stimulator. Expansion of the membrane started 15 ms after trigger onset and peaked 20 ms later (i.e., 35 ms after trigger onset). The total duration of membrane movement was around 100 ms. This stimulation method has been used previously in a number of EEG and MEG studies (Pihko and Lauronen, 2004; Saby et al., 2015; Shen et al., 2018).

#### Task Partner

**FIGURE 1:**
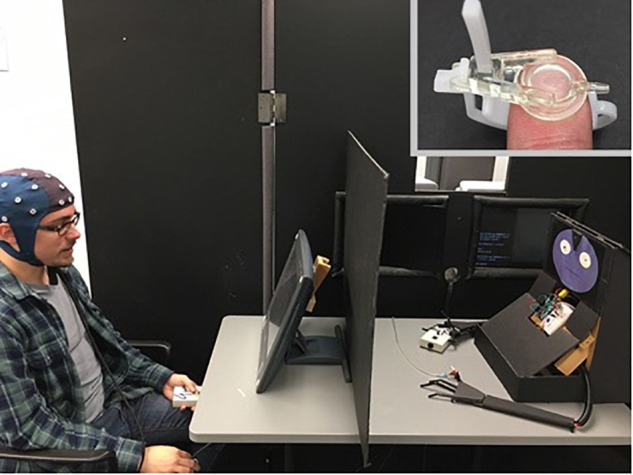
Experimental setup during the robot condition. Participants responded to visual stimuli presented on the monitor. The robot used for this study is on the right side of the barrier. The embedded image in the top right shows how the stimulation device is attached to the index finger of the participant. Written informed consent was obtained from the individual on the left for publication of this image.

Prior to participating in the experimental procedure, participants were shown a demonstration of the robot that they would be interacting with. The robot was implemented via an Arduino UNO board. The robot was comprised of left and right “hands,” a torso, and a head (see Figure 1). The left hand contained a single haptic sensor; the right hand had a single point of articulation at the position of the index finger, which was movable via a servo embedded within the hand. The index finger was programmed to move downward to touch the surface in front of it in response to either a flash of light, or a touch to the left hand during the demonstration. Two red LEDs served as the “eyes,” while a small photoreceptor was placed between these LEDs. Participants were asked to shine a light from a cellphone over the photoreceptor of the robot, which triggered movement of the right index finger. Participants were shown a small LED light and were told that during the study, the robot would know when it was its turn to press the button based on this LED flashing toward its photoreceptor. All participants were given the same introduction to the robot, and told they would be carrying out a joint task involving reciprocal tactile stimulation.

Following the demonstration of the robot, participants were all given the same introduction to their human task partners, and told they would be carrying out the same joint task as with the robot. Participants were shown that the human partner would see the same visual cues as them, wear the same inflatable membrane, and press an identical button to initiate tactile stimulation.

### Design, Task, and Procedure

#### Procedure

Participants were seated 60 cm from a flat panel monitor (40 cm viewable), on which visual cues relating to the onset of tactile stimulation were presented. Participants held a small box in their left hand on which a single response button was mounted. Seated across from the participant was either a human partner or the robot, depending on condition and order. During each block, the participant was aware whether their partner was a human or the robot, but could not see them (Figure 1): Participants were separated from their task partner by a divider, in order to control for visual influences during data collection. To mask any subtle sounds associated with delivery of the tactile stimuli, participants wore earplugs during EEG collection, and ambient white noise was broadcast in the testing room.

#### Task Conditions

**FIGURE 2:**
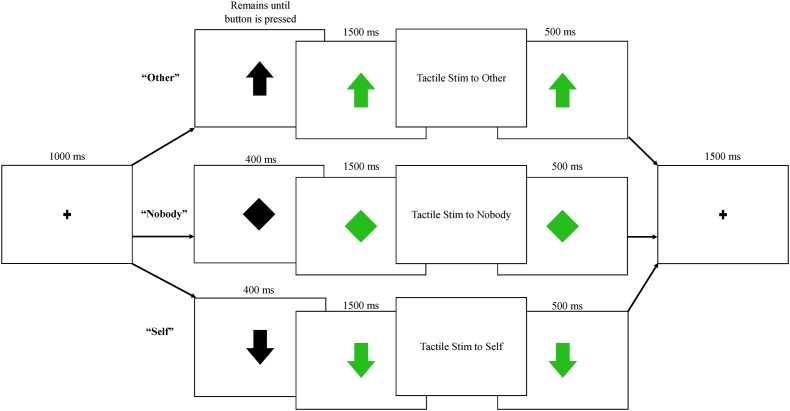
Each trial began with a fixation point, followed by one of three possible trials: *other*, in which subjects pressed a button and their partner received tactile stimulation; *nobody*, in which no one pressed a button or received stimulation; *self*, in which participants were told their partner was pressing a button, triggering tactile stimulation to the participant’s right index finger. All trials were presented equally frequently across human and robot conditions.

Participants engaged in three blocks of trials with a human partner and three blocks of trials with a robot partner. All blocks within a condition (human/robot partner) occurred together, and the order of presentation (human first/robot first) was counterbalanced between participants. Prior to beginning the protocol, a practice trial was conducted by an experimenter, who demonstrated each of the three trial types shown in Figure 2: (1) *Nobody* trials: during these trials, an initial fixation point was replaced with a black diamond, which then turned green indicating that a tactile event was being sent to “nobody.” Neither the participant nor the partner were required to press a button, and an air pulse was sent to an inflatable membrane (not attached to anyone or anything) in the testing room; (2) *Self* trials: during these trials, the fixation point was replaced by a black arrow facing downward, indicating that the participant could expect tactile stimulation to delivered to his or her right hand following a button press by the partner. Participants were told that the arrow turns green when their partner presses the button. Importantly, the partner (human or robot) was not actually triggering stimulation; the arrow turned green at a fixed interval of 400 ms following the black arrow across both conditions. 1500 ms after the arrow turned green, tactile stimulation was delivered to the participant’s finger. The trial timing was held constant across conditions, in order to keep the human/robot conditions as similar as possible aside from the type of partner; (3) *Other* trials: During these trials, a black arrow facing upward replaced the fixation, indicating that the participant could now press the button with his or her left hand, which then triggered the arrow to turn green and initiated the tactile pulse to be delivered to the partner’s hand 1500 ms later (See Figure 2).

Each of these three trial types was presented 80 times within each condition (human/robot), resulting in six different conditions and a total of 480 trials; since the *nobody* conditions did not differ in any way between partner types, these were collapsed into a single condition, resulting in five conditions for analyses: *nobody*: tactile stimulation is triggered and felt by no one; *self-human*: tactile stimulation is sent from a human partner to the participant; *self-robot*: tactile stimulation is sent from a robot partner to the participant; *other-human*: tactile stimulation is sent from the participant to a human partner; *other-robot*: tactile stimulation is sent from the participant to a robot partner. Trials within the *nobody* and *self* conditions were 4900 ms in length, while trials in the *other* conditions varied in duration due to variation in reaction time for the button press. The mean reaction time for the button press was 280 ms, resulting in an average trial length of 4780 ms for this condition. Data collection lasted approximately 45 min, including breaks between each block. *Nobody* trials were randomly presented during the blocks, while self and other trials were always presented in a self-other-self or other-self-other fashion, as a way to keep the reciprocal nature of the task salient. The presentation of these three trial units was randomized across all blocks.

Following EEG collection, a brief questionnaire was given as a manipulation check, consisting of 13 questions about their performance with the robot partner, rated on a Likert scale from 1 (strongly disagree) to 5 (strongly agree). Overall, participants believed that their human partner chose when to push the button (*M* = 4.85, *SD* = 0.95), while participants were less likely to agree that the robot partner chose when to push the button (*M* = 3.00, *SD* = 1.3).

#### Joint Task

Participants were given instructions for completing the tactile attention task in cooperation with their partners during each of the six blocks. Within each block, there were trials during which the participant received two tactile pulses rather than one. Participants were instructed to count the number of these double pulses within each block. Before beginning the experiment, each participant received practice in distinguishing the double pulses from the regular single pulses. For each block, a predetermined number of double pulses was sent to the partner, and between 3 to 12 pulses were sent to the participant. After each block, an experimenter entered the room and asked the participant how many double pulses he or she had felt, and then either asked the human partner or checked a small LCD screen on the robot. The respective totals were summed and compared to a total that was unknown to the participant, but was known to the experimenter. The researcher would report the correct total, and as appropriate, would state the number of missed double pulses. Trials with double pulses were excluded from EEG analyses. Participants’ mean performance on the attentional task of detecting double pulses was 92%; performance did not differ between condition or order.

### Data Acquisition

The EEG signal was acquired from 32 electrodes secured in a Lycra stretch cap (ANT Neuro, Germany) according to the International 10–20 format. Each electrode casing was filled with a small amount of conductive gel. Preparation of the EEG cap took place after participants had been given the demonstration of the robot, before the practice trials. The EEG signals were collected referenced to Cz with an AFz ground, and were re-referenced offline to the average of the left and right mastoids. Eye blinks were monitored via EOG electrodes placed above and below the left eye. Scalp impedance at each electrode site was kept under 25 kΩ. All EEG and EOG signals were amplified by optically isolated, high input impedance (>1 GΩ) bio amplifiers from SA Instrumentation (San Diego, CA, United States) and were digitized using a 16-bit A/D converter (+/- 2.5 V input range) at a sampling rate of 512 Hz using Snap-Master data acquisition software (HEM Data Corp., Southfield, MI, United States). Hardware filter settings were 0.1 Hz (high-pass) and 100 Hz (low-pass) with a 12 dB/octave rolloff. Bioamplifier gain was 4000 for the EEG channels and 1000 for the EOG channels.

### Data Analysis

#### Preprocessing of EEG Data

Electroencephalogram analysis was performed using the EEGLAB 13.6.5b toolbox (Delorme and Makeig, 2004) implemented in MATLAB. Epochs were extracted from the continuous EEG data. For the analysis of the *nobody/self* conditions, each extracted epoch was 3500 ms in duration, beginning 600 ms before visual cue onset and ending 1000 ms after the onset of the tactile stimulus. For analysis of the *other* conditions, each epoch was time-locked to the moment when the participant pressed the button, which triggered stimulation to their participant that occurred 1500 ms later. For these trials, analysis began at -2900 ms relative to the 0 ms point of tactile stimulation delivery, with a baseline period of the 500 ms prior to the visual cue, during the display of the fixation point.

Independent component analysis was conducted to remove eye movement artifacts (Hoffmann and Falkenstein, 2008). Visual inspection of the EEG signal was used to reject epochs containing movement artifact. Across all participants, 92.43% of trials were retained. There was no significant difference in the number of rejected epochs between trial type, *p* = 0.387.

#### Time Frequency Analysis

Time-frequency decompositions of single trial data were conducted using event-related spectral perturbation (ERSP) analysis (Makeig, 1993), for a 2500 ms window that ran from -2000 ms prior to the onset of the tactile stimulus to 500 ms after tactile stimulation onset. ERSP was computed using a Morlet wavelet decomposition over a frequency range of 5–30 Hz, with 100 overlapping windows starting with a 3-cycle wavelet at the lowest frequency. Event-related desynchronization (ERD) was taken as an ERSP decrease relative to the baseline.

#### Statistical Analyses

Mean ERSP in the mu band (8–13 Hz) over the 1000 ms epoch leading up to the onset of the tactile stimulus was computed for centroparietal electrodes overlying the contralateral (CP1, CP5, P3 and C3) and ipsilateral (CP2, CP6, P4 and C4) sensorimotor cortex. In order to assess anticipatory effects induced by the different conditions over this region of interest, mean ERSP was submitted to a 3 × 2 repeated-measures ANOVA involving condition (*human, robot, nobody*) and hemisphere (contralateral, ipsilateral). To confirm whether any condition effects were regionally specific, we also executed a mass univariate analysis comparing alpha ERSP amplitudes between conditions for each of the 32 electrodes.

In order to assess differences in beta band responses following the button press by the participants, mean beta (14–22 Hz) ERSP over the 1000 ms epoch following the button press was compared between the two relevant conditions (*other-human, other-robot*) across three target electrode regions within a 2 × 3 repeated-measures ANOVA. The beta band of 14–22 Hz was chosen based on previous research on post-movement beta responses, in which modulation of power is most frequently seen in the 15–20 Hz range (Pfurtscheller, 2001). The three regions encompassed frontocentral (Fz, FC1, FC2, Cz) and centroparietal electrodes overlying the contralateral (CP1, CP5, P3 and C3) and ipsilateral (CP2, CP6, P4 and C4) sensorimotor cortex. To confirm whether any condition differences were regionally specific, we also executed a mass univariate analysis comparing beta ERSP amplitudes between conditions at each of the 32 electrodes.

## Results

### Tactile Anticipation

#### Mu Rhythm (8–13 Hz)

The ANOVA for mu ERSP indicated significant main effects of condition, *F*(2,18) = 18.63, *p* < 0.001 and hemisphere, *F*(1,19) = 22.88, *p* < 0.001. Further, the ANOVA indicated a significant interaction of condition and hemisphere, *F*(1,19) = 14.32, *p* < 0.001. Follow-up analyses indicated that mu ERSP over the contralateral (i.e., left) centroparietal region was significantly reduced (indicating greater desynchronization) when participants expected tactile stimulation to self (whether initiated by a *robot*, *M* = -0.89, *SD* = 0.701, or a *human*, *M* = -1.02, *SD* = 0.635), compared to trials when no tactile stimulation was expected (*nobody*, *M* = -0.94, *SD* = 0.411). There was no significant difference between conditions over the ipsilateral centroparietal region.

**FIGURE 3:**
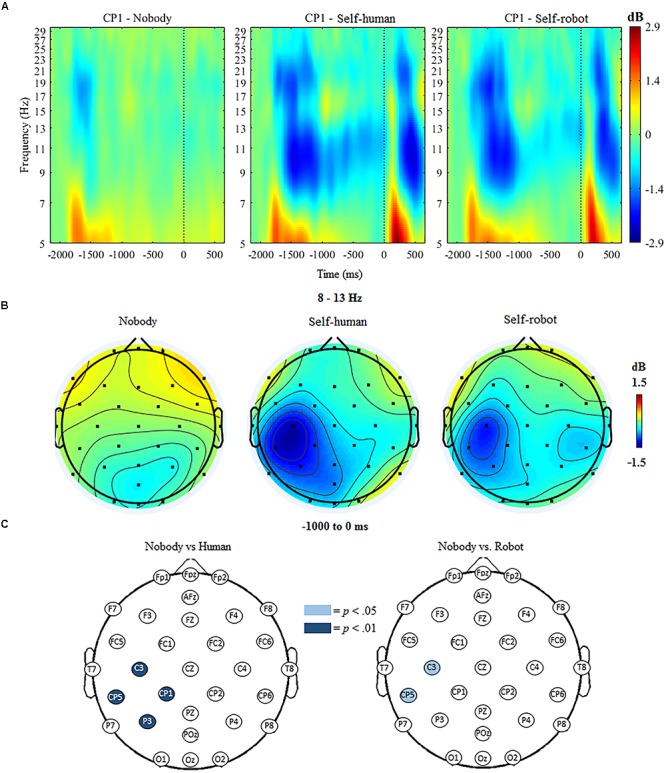
**(A)** Event-related spectral perturbation (ERSP) at electrode CP1 during *nobody* trials and *self* trials (in human/robot conditions), with alpha ERD in the time window during tactile anticipation. **(B)** Topographic maps showing activity in the mu/alpha (8–13 Hz) band during anticipation of tactile stimulation across conditions. **(C)** Differences in 8–13 Hz desynchronization between *nobody* and human-*self* and *nobody* and robot-*self* trials, respectively. Significantly different electrodes are highlighted, showing differences in the mu rhythm over central sites based on paired samples *t*-tests. The nose is located at the top of the scalp map.

The mass univariate analyses confirmed regional specificity of effects by showing that anticipatory mu ERD was significantly different at the *p* < 0.01 threshold at various centroparietal electrodes, but not over other scalp regions, when comparing stimulation from the *human* to the *nobody* condition. These differences were apparent at C3, *t*(18) = 5.31, *p* < 0.001, CP1, *t*(18) = 3.56, *p* = 0.002, P3, *t*(18) = 3.98, *p* < 0.001, and CP5, *t*(18) = 4.65, *p* < 0.001, such that mu desynchronization at these electrodes was greater for the *human* condition compared to the *nobody* condition. Compared with the *nobody* condition, there was also significantly greater mu desynchronization in the *robot* condition at C3, *t*(18) = 2.79, *p* = 0.012, and CP5, *t*(18) = 2.67, *p* = 0.015 at the *p* < 0.05 threshold (see Figure 3). For the direct comparison of mu ERSP in relation to the source of stimulation to the self (*human vs. robot*), there was only a marginal difference in amplitude observed at one electrode (CP1), *F*(1,19) = 2.081, *p* = 0.061; ERSP at all other electrodes did not differ significantly between the human and robot conditions.

### Execution of Action

#### Behavioral Measures

Mean reaction time for participants’ button presses following the visual cue did not differ between conditions (mean RT with human partner = 278.68 ms, *SD* = 16.94; mean RT with robot partner = 282.62, *SD* = 14.22), *t*(19) = -0.74, *p* = 0.463.

#### Beta Band (14–22 Hz)

The ANOVA for beta ERSP indicated significant main effects of condition, *F*(1,18) = 7.45, *p* < 0.001 and region, *F*(1,19) = 21.96, *p* < 0.001. The ANOVA further indicated a significant interaction of condition and region, *F*(1,19) = 14.32, *p* < 0.001. Follow up analyses indicated that there was a significantly greater beta ERSP after the button press over frontocentral regions when participants delivered tactile stimulation to a robot (*M* = 1.02, *SD* = 0.67), compared to trials where participants delivered stimulation to a human (*M* = 0.02, *SD* = 0.59). There were no significant differences between conditions at centroparietal sites.

**FIGURE 4:**
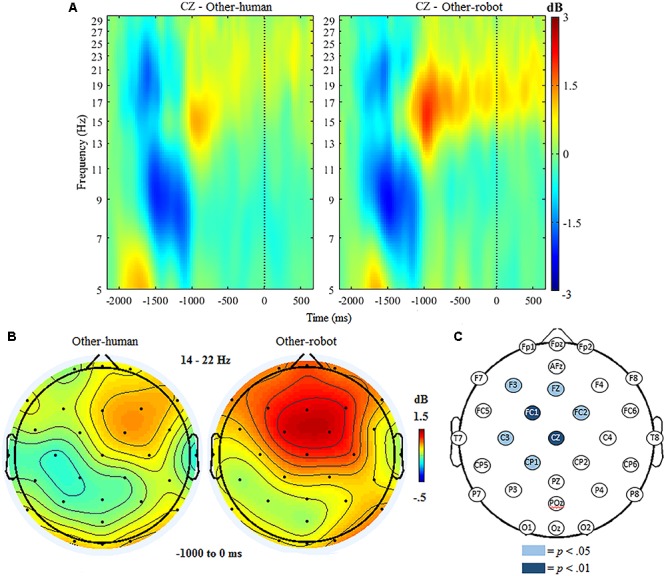
**(A)** Event-related spectral perturbation at electrode Cz during *other* trials, with beta ERS present the time window following the button press (–1000 to 0 ms). *Human-other* and *robot-other* conditions are shown separately. **(B)** Topographic maps showing condition differences for post-button press activity in the beta band (14–22 Hz) from –1000 to 0 ms, with 0 ms being time locked to the participant’s button press. **(C)** Differences in beta power increase between human and robot conditions in the *other* trials. Significantly different electrodes are highlighted, showing differences in beta ERSP over central sites based on paired samples *t*-tests. The nose is located at the top of the scalp map.

Mass univariate analyses further indicated that the above effect was specific to frontocentral sites, with beta ERSP being significantly greater in *other-robot* trials than for *other-human* trials at electrodes FC1, *t*(18) = 6.31, *p* < 0.001, and Cz, *t*(18) = 5.95, *p* < 0.001, at the *p* < 0.01 threshold. Beta ERSP was significantly greater in *other-robot* trials at F3, *t*(18) = 2.80, *p* = 0.011, Fz, *t*(18) = 2.68 *p* = 0.015, FC2, *t*(18) = 2.60 *p* = 0.018, CP1, *t*(18) = 2.53, *p* = 0.021, and C3, *t*(18) = 2.49, *p* = 0.022) at the *p* < 0.05 threshold (see Figure 4).

## Discussion

We investigated sensorimotor oscillations during a reciprocal touch paradigm, using EEG measures to compare aspects of brain oscillatory responses to receiving and initiating tactile stimulation during a joint task involving either a human or robot partner. Our specific questions were twofold: First, whether desynchronization of the sensorimotor mu rhythm during anticipation of tactile stimulation differed according to the perceived origin of the stimulation (as being initiated by a human vs. a robot). Second, whether EEG beta band responses to the act of initiating delivery of a tactile stimulus to another entity differed according to whether that entity is a human or a robot.

### Anticipation of Tactile Stimulation

In line with previous research (Shen et al., 2017), there was a clear desynchronization of the EEG mu rhythm over the contralateral central region during the anticipation of tactile stimulation to self. A similar desynchronization was not present during the “nobody” condition in which a cue was present and a stimulus was triggered, but the stimulus was not directed toward anyone. In terms of the central question, we found little evidence for a differential modulation of mu rhythm activity when participants anticipated tactile stimulation that they believed was initiated by a button press from a robot or a human partner. The extent of anticipatory mu desynchronization did not meaningfully differ in amplitude when the source of the tactile stimulation was the action of a human as opposed to a robot. Given that anticipatory mu desynchronization is considered an index of selective attention in the tactile modality (van Ede et al., 2012; Weiss et al., 2018), these results suggest that participants were equally attentive in monitoring for upcoming tactile stimulation from human and robot partners. While the directions of the means suggested that mu ERD was somewhat greater when participants expected stimulation initiated by a human rather than a robot partner, only trend-level differences in amplitude were apparent, and only at one electrode site.

One strength of our task protocol was that visual cues were constant across conditions, in order to isolate the influence of participant’s beliefs about the nature of their partner on the brain responses during the task. However, it is also possible that the salience of the manipulation could be increased by allowing participants to observe the human or robot partner press the button. The subtle differences we observed can be further investigated by providing participants with contingent visual information about the nature of the partner, or providing a more “social” rather than physical interaction with the robot partner.

Although it is understood that the extent of anticipatory mu rhythm desynchronization is related to subsequent perceptual processing of the target stimulus (Zhang and Ding, 2010), little is known about the determinants of the anticipatory mu response, including individual differences. There remains sustained interest in the neural processes underlying the mapping of somatosensory experience from one own body to that of another (Keysers et al., 2010; Marshall and Meltzoff, 2015). One study found no clear evidence for mu desynchronization during the anticipation of tactile stimulation delivered to another person (Shen et al., 2017), and we did not examine this question here. Instead, the current study took a novel approach by examining how the perceived origin of tactile stimulation modulated the anticipatory mu response to self. With this in mind, we considered anticipatory processes in the context of a sustained interactive task, which also allowed us to examine processes related to the sending of tactile stimulation to the partner.

### Execution of Action

One well-studied electrophysiological correlate of action production (particularly finger movements, such as a button press) is the beta rebound response, which takes the form of an increase in beta band power after a brief reduction of power immediately following the action (Cheyne, 2013). Here we found modulation of the beta rebound response in the period after participants initiated tactile stimulation to a partner, prior to the actual delivery of the tactile stimulus. When participants initiated tactile stimulation that was directed toward a robot, there was greater beta ERS across central and frontal electrode sites. The exact function and mechanism of beta ERS following action production is not entirely understood, but it is believed to partly reflect motor inhibition (Heinrichs-Graham et al., 2017). In certain contexts, post-movement beta ERS relates to increases in cortical deactivation (Pfurtscheller, 2001), particularly in the lower beta (14–22 Hz) range used in the current study. The greatest level of beta ERS was seen over frontoparietal sites, which is expected based on the localization of post-movement ERS to motor cortex (Jurkiewicz et al., 2006). The meaning of these differences between conditions in beta ERS still needs to be elucidated. One line of reasoning relates to the idea that reduced beta band activity increases the capability for cognitive and motor flexibility in terms of upcoming or future responses (Engel and Fries, 2010). As such, enhancement of beta band activity (i.e., a larger rebound effect) in the context of HRI may reflect the perceived greater predictability of robot compared to human partners. Further work can examine this speculation as well as investigate other possible influences on the beta response (e.g., differing button press force between conditions).

In behavioral studies within the domain of HRI, reactions to robots vary greatly across studies, due in part to the wide range of robotic forms implemented across this area of research (Hoffmann et al., 2010). In some contexts, artificial agents may be more engaging than human counterparts (Gratch et al., 2007), but within most natural contexts, people tend to prefer the company of humans to machines. Increased beta ERS while interacting with the robot could reflect a different attentional state, or a decrease in uncertainty regarding the outcome of the present action (Engel and Fries, 2010). This speculation warrants further investigation, as do alternative explanations for the differential beta rebound responses. The nature of the present paradigm was limited in how much it immersed the participant in interactions with the partner; beyond the initial introductions, participants only interacted with the human or robot partner through the delivery of tactile stimulation, without any visual, auditory, or direct physical contact. The non-visual nature of the present study was intentional, given the strong visual effects found in previous research derived from aesthetic differences between human and robotic stimuli (Press, 2011). Furthermore, the embodiment of the robot in the current study was somewhat limited. The addition of different sensory modalities and the use of more sophisticated robot platforms could help to develop a richer picture of brain responses during interactions with robots.

### Implications

The results of this study provide some of the first evidence for differences in attentional and tactile processing when interacting with human and robotic partners. Past research on HRI has focused largely on differences in physical appearance and abilities (Hoffmann et al., 2010; Paauwe et al., 2015; Strait et al., 2017), while the present study removed visual information from the experimental procedure; differences between conditions are therefore likely to be the result of a participant’s beliefs, rather than visual input during the task. Previous work in HRI has begun to identify factors which influence how people respond to touch from robots (Nakagawa et al., 2011; Wullenkord et al., 2016), and future work on the cognitive neuroscience of HRI will need to incorporate these factors into the design of robots used in this line of research. Research in this area could also draw on emerging work in the area of “sociomotor action control” which has clear implications for progress in HRI (Kunde et al., 2017).

Future work with this and similar paradigms will continue to shed light on sensory processing in the context of stimuli that are delivered by a non-human agent. A potential follow-up to the present study could include an additional condition in which neither a human nor robot is present during tactile stimulation to the participant; such a condition would allow further exploration of the effects of other agents on sensory processing. Given the controlled nature of the present study, the tasks has little resemblance to a typical social interaction. Further studies with a similar paradigm could be situated in naturalistic contexts, such as physical therapy, wherein touch is an integral and natural part of the interaction. Additional work could also examine differences between protocols that involve passive touch (as in the task used in the current study) and the more active kinds of touch that characterizes typical human–environment interactions.

Within research on brain-computer interfaces (BCI), sensorimotor oscillations are most frequently targeted as a source of input to control various machines or devices (Yuan and He, 2014). In this line of work, the beta rhythm has been targeted most frequently, specifically post-movement beta rebound, due to the well-timed relation between motor movements and the corollary oscillations measurable through EEG (Pfurtscheller and Solis-Escalante, 2009). These oscillations are of particular interest for BCI researchers due to the range of human behaviors which can engender them, with and without overt motor movement. Through examination of sensorimotor activity in response to the use of BCI, feedback loops can be created which form a sort of continuous connection to brain-controlled machines (Neuper et al., 2009), allowing for the control of machines through motor imagery alone (Pfurtscheller and Neuper, 2010). The present study can inform the creation of BCI platforms by showing how beta rebound, and sensorimotor oscillations in general, may be influenced by the nature of the machine being acted upon. Follow-up work on this issue could be conducted across a range of different types of machines from humanoid robots and androids to simple mechanical machines. Algorithms that are robust to psychological perturbations on sensorimotor rhythms would be ideal in the application to BCI.

In addition to helping us understand how to better integrate robots in social contexts, a social-cognitive neuroscience approach to robotics can provide insights beyond the field of HRI (Broadbent, 2017). Robots provide a unique control in social paradigms, as various levels of intentionality, autonomy, and humanoid appearance can be manipulated. Human reactions to robotic bodies varies greatly depending on the nature of the machine and context in which it is experienced (Chaminade and Cheng, 2009), but there appears to be something unique about the way in which we process information about a thing when that thing is a fellow human (Saygin et al., 2011; Urgen et al., 2013).

## Ethics Statement

This study was carried out in accordance with the recommendations of the Institutional Review Board of Temple University with written informed consent from all subjects. All subjects gave written informed consent in accordance with the Declaration of Helsinki. The protocol was approved by the Institutional Review Board of Temple University. The writing of this article was supported in part by an award from NSF (BCS-1460889).

## Author Contributions

NS developed the reciprocal touch paradigm and study design, designed and constructed the robot, carried out data acquisition and analysis, wrote the initial draft of manuscript, carried out subsequent editing and formalization, and created figures. SW contributed to study development, data acquisition and analysis, assisted in drafting and editing of manuscript, contributed analytic tools for analysis of EEG data, reviewed and described the literature on the EEG alpha rhythm. PM assisted with the inception of the study, advised on experimental design, and edited the manuscript.

## Conflict of Interest Statement

The authors declare that the research was conducted in the absence of any commercial or financial relationships that could be construed as a potential conflict of interest.
